# Characterisation of tumor‐infiltrating gamma‐delta T cells in human colorectal cancer with MHC‐I loss

**DOI:** 10.1002/cti2.70097

**Published:** 2026-05-12

**Authors:** Tianming A Li, Luke T Quigley, Kok Fei Chan, David S Williams, Lisa A Mielke, Andreas Behren, Jessica Da Gama Duarte

**Affiliations:** ^1^ Olivia Newton‐John Cancer Research Institute Heidelberg VIC Australia; ^2^ School of Cancer Medicine La Trobe University Heidelberg VIC Australia; ^3^ Department of Cancer Medicine, School of Translational Medicine Monash University Melbourne VIC Australia; ^4^ Department of Pathology Austin Health Heidelberg VIC Australia; ^5^ Department of Medicine University of Melbourne Parkville VIC Australia

**Keywords:** colorectal cancer, MHC class I loss, multispectral immunohistochemistry, γδ T cells

## Abstract

**Objectives:**

Gamma‐delta (γδ) T cells have been associated with favorable prognoses across several malignancies, underscoring their potential as targets for novel immunotherapies. These unconventional T lymphocytes exhibit an intrinsic tropism for the tumor microenvironment, largely driven by their capacity to recognize stress‐induced antigens characteristic of metabolically dysregulated tumors. Unlike the mechanism governing conventional cytotoxic CD8^+^ alpha‐beta (αβ) T cells, γδ T‐cell receptors can engage tumor cell moieties independent of major histocompatibility complex class‐I (MHC‐I) and human leukocyte class‐I molecules. Therefore, γδ T cells may have a pivotal role in the immune response to beta‐2 microglobulin‐mutated MHC‐I negative (MHC‐I^−^) colorectal cancers (CRCs) with deficient mismatch repair.

**Methods:**

To determine whether γδ T‐cell mobilisation extends to diverse aetiologies of MHC‐I loss, we used multispectral immunohistochemistry to stain 150 stage I‐IV primary CRC tissues, across eight tissue microarrays.

**Results:**

Our investigation revealed MHC‐I loss in ~30% of CRC primary tumors across all disease stages, with a notable increase in stromal γδ T‐cell frequency and activation status among stage III cases. Importantly, these findings extend previous observations largely confined to mismatch repair–deficient CRC by demonstrating that stromal γδ T‐cell enrichment associated with MHC‐I loss also occurs in mismatch repair–proficient tumors, suggesting a broader role for γδ T cells in immune surveillance of MHC‐I–deficient CRC.

**Conclusion:**

These data highlight the potential of γδ T cells in counteracting immune evasive MHC‐I^−^ tumors, thereby offering a robust rationale for their strategic deployment in next‐generation immunotherapy regimens.

## Introduction

Colorectal cancer (CRC) patients with microsatellite instability‐high (MSI‐H) and deficient DNA mismatch repair (dMMR) tumors have demonstrated robust clinical responses to various immune checkpoint blockade (ICB) therapies.^1^, ^2^, ^3^, ^4^, ^5^, ^6^, ^7^ dMMR/MSI‐H tumors constitute approximately 10–20% of all CRC cases,^8^, ^9^, ^10^ with diagnosis determined via the immunohistochemical assessment of MMR protein expression and molecular testing of tumor DNA for MSI status.^11^ Commensurate with other cancers with high tumor mutational burdens,^12^ dMMR CRCs accumulate diverse neoantigen repertoires likely to trigger potent immune responses and limit tumor progression.^13^

However, a significant subset of dMMR CRCs (20–60%) acquire mutations that affect the expression of major histocompatibility complex class‐I (MHC‐I) molecules,^14^ hindering tumor recognition by antigen‐experienced CD8^+^ T cells. Causes of MHC class‐I loss (MHC‐I^−^) in CRC tumors can arise from all stages of the central dogma, including direct genetic mutations, epigenetic modifications, transcriptional variations and translational changes.^14^ These aberrations consistently result in reduced immune surveillance and dampened anti‐tumor CD8^+^ T‐cell cytotoxicity, causing tissue reorganisation and the exclusion of specific immune cell subsets from the tumor region.^15^

Analysis of MHC‐I expression primarily relies on immunohistochemistry techniques to probe surface molecules, though studies have traditionally differed by protocols and thresholds to determine downregulation and loss.^16^, ^17^, ^18^, ^19^, ^20^, ^21^, ^22^, ^23^, ^24^, ^25^ On a genomic level, mutational analyses have focussed on crucial components of the MHC‐I antigen presentation pathway, with particular emphasis on beta‐2‐microglobulin (β2M),^26^ a component essential for the proper folding and surface expression of human leukocyte antigen class‐I (HLA‐I).

A compelling paradox has emerged from clinical trials involving ICB in CRC.^2^, ^27^, ^28^, ^29^, ^30^, ^31^, ^32^, ^33^, ^34^ Specifically, β2M‐deficient CRC lacking MHC‐I expression has shown clinical benefit from ICB despite the theoretical absence of a robust CD8^+^ T‐cell immune response. This unexpected finding pointed towards the contribution of alternative immune cell subsets,^35^ notably gamma‐delta (γδ) T cells, which have previously been identified as a favorable prognostic marker across solid tumors,^36^ and are associated with improved 5‐year survival in early‐stage CRC patients.^37^, ^38^

A recent study by De Vries *et al*.^39^ implicated Vδ1^+^ and Vδ3^+^ γδ T cells as principal agents of tumor clearance in β2M‐mutated MHC‐I defective CRC tumors. Unlike circulating γδ T cells that are composed primarily of the Vδ2^+^ subset,^40^ Vδ1^+^ cells are predominantly localized within mucosal and epithelial tissues, such as the colon, and are often enriched in the tumor microenvironment, particularly those of epithelial origin.^41^ Within the tumor microenvironment, γδ T‐cell subsets exhibit heightened cytotoxicity markers and elevated PD‐1 expression.^42^ Unlike conventional alpha‐beta (αβ) T cells,^43^ the activation of γδ T cells occurs independently of MHC‐I molecules.^44^ It is speculated that this immune response is mediated through surface receptors such as natural killer group 2D (NKG2D) and DNAX accessory molecule‐1 (DNAM‐1) that recognize stress‐induced ligands—including MHC‐I chain‐related proteins A and B (MICA/B) and UL16‐binding proteins (ULBPs).^40^, ^45^ These activation pathways are particularly relevant in tumors that have lost MHC‐I expression, as they provide an alternative recognition mechanism to overcome immune evasion.

While the involvement of γδ T cells in dMMR CRCs is becoming clear, their contribution in proficient DNA mismatch repair (pMMR) tumors with MHC‐I loss remains largely unknown.^46^ This is an important distinction, as pMMR tumors constitute the majority of CRC cases and are typically unresponsive to ICB.^1^ Whether similar γδ T‐cell responses occur in pMMR CRCs with MHC‐I loss, and how these cells are spatially distributed within the tumor microenvironment, remains poorly defined. To address this gap in the literature, the spatial distribution of γδ T cells was interrogated within the CRC tumor microenvironment. Opal™ fluorophore‐tyramide signal amplification (TSA) multispectral immunohistochemistry staining was performed on a large cohort of 192 CRC tumor tissues to probe for immune infiltrates and their correlation with MHC‐I expression at various disease stages. A custom antibody panel consisting of TCRδ, CD3, PD‐1, MHC‐I, pan‐cytokeratin (PanCK) and DAPI was used to stain tissue microarrays (TMAs). Remarkably, a significant increase in stromal γδ T cells was identified compared with their tumor‐infiltrating counterparts in MHC‐I^−^ stage III CRCs. Building upon prior studies, this observation extends to the pMMR CRC subset, further emphasising the potential for γδ T cells to contribute to an anti‐tumor response when conventional αβ T‐cell immunity is compromised.

## Results

### Epithelial MHC‐I expression varies in CRC

**Figure 1:**
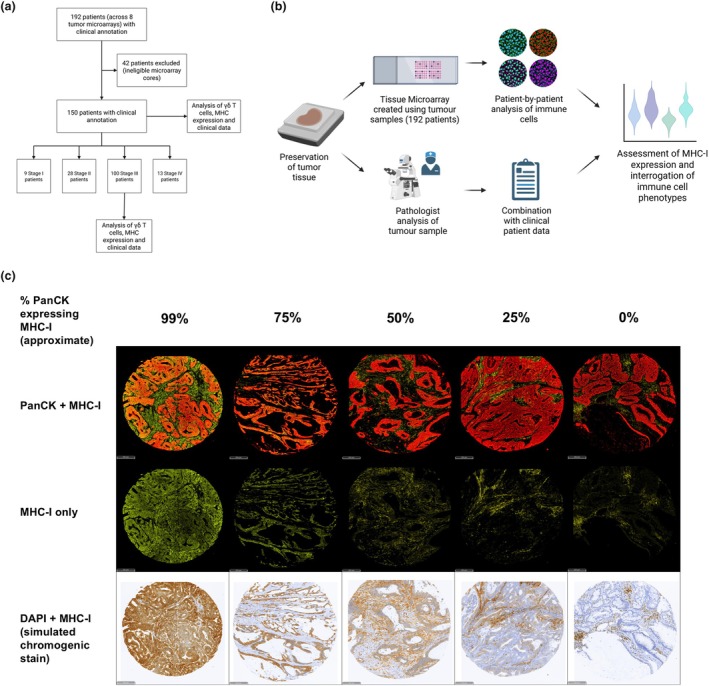
Study overview and epithelial MHC‐I expression criteria. MHC‐I expression was quantified in individual CRC samples by calculating the percentage of PanCK^+^ cells co‐staining with MHC‐I. **(a)** Primary tumor tissue from 192 CRC patients was included in eight TMAs. Core inclusion criterion was based on the presence of tumor regions, and exclusion criteria included defects in sample quality, deparaffinisation and staining processes. Cores from a total of 150 CRC patients were included in the subsequent cohort‐ or stage‐specific analyses. **(b)** TMA sections were stained, imaged, and analysed using histopathology software, and combined with clinical and histopathology data for downstream analysis. **(c)** Spectrum of MHC‐I expression across different patient tumors. A simulated chromogenic stain for the same core is included for each column. Scale bars indicate 200 μm. Images were taken at 20× magnification. Graphs were created using Biorender.

The study cohort selection and analysis workflow are depicted in Figure 1a and b, respectively, with detailed patient characteristics provided in Table 1. To illustrate the observed heterogeneity in MHC‐I expression, representative patient cores with quantified MHC‐I expression levels, approximating 99%, 75%, 50%, 25% and 0% of PanCK+ cells expressing MHC‐I, are shown in Figure 1c. Notably, MHC‐I expression remained consistent within stromal (PanCK‐) regions across samples, while regions co‐staining with PanCK exhibited variability across patients. Chromogenic staining to simulate traditional immunohistochemistry is also presented in the bottom row of Figure 1c for MHC‐I and nuclear DAPI, consistent with stains reported in previous studies on MHC‐I or β2M expression.^14^, ^39^, ^47^

**Table 1:** Characteristics of the patient cohort used in this study

Characteristic	All stages (*n* = 150)	Stage 1 (*n* = 9)	Stage 2 (*n* = 28)	Stage 3 (*n* = 100)	Stage 4 (*n* = 13)
Age					
≥ 75	54 (36.0)	4 (44.4)	11 (39.3)	35 (35.0)	4 (30.8)
65–74	40 (26.7)	2 (22.2)	8 (28.6)	26 (26.0)	4 (30.8)
50–64	37 (24.7)	3 (33.3)	6 (21.4)	26 (26.0)	2 (15.4)
< 50	18 (12.0)		3 (10.7)	12 (12.0)	3 (23.)
Not reported	1 (0.6)			1 (1.0)	
Sex					
Male	30 (20.0)	2 (22.2)	10 (35.7)	15 (15.0)	3 (23.1)
Female	52 (34.7)	7 (77.8)	18 (64.3)	17 (17.0)	10 (76.9)
Not reported	68 (45.3)			68 (68.0)	
HLA class I					
High expression (HLA‐I^+^)	101 (67.3)	6 (66.7)	18 (64.3)	67 (67.0)	10 (76.9)
Low expression (HLA‐I^−^)	49 (32.7)	3 (33.3)	10 (35.7)	33 (33.0)	3 (23.1)
MMR status					
Proficient (pMMR)	102 (68.0)	4 (44.4)	8 (28.6)	80 (80.0)	10 (76.9)
Deficient (dMMR)	48 (32.0)	5 (55.6)	20 (71.4)	20 (20.0)	3 (23.1)
BRAF status					
Wild type	111 (74.0)	4 (44.4)	18 (64.3)	77 (77.0)	12 (92.3)
Mutant	39 (26.0)	5 (55.6)	10 (35.7)	23 (23.0)	1 (7.7)
TILs (Pathologist scored)					
Low (< 2 per hpf)	88 (58.7)	3 (33.3)	5 (17.9)	70 (70.0)	10 (76.9)
High (≥ 2 per hpf)	62 (41.3)	6 (66.7)	23 (82.1)	30 (30.0)	3 (23.1)
Tumor site					
Left colon	31 (20.7)	2 (22.2)	2 (7.1)	24 (24.0)	3 (23.1)
Right colon	102 (68.0)	7 (77.8)	24 (85.8)	61 (61.0)	10 (76.9)
Rectum	15 (10.0)		2 (7.1)	13 (13.0)	
Not reported	2 (1.3)			2 (2.0)	
Grade summary					
Low grade	102 (68.0)	7 (77.8)	19 (67.9)	67 (67.0)	9 (69.2)
High grade	47 (31.3)	2 (22.2)	9 (32.1)	32 (32.0)	4 (30.8)
Not reported	1 (0.7)			1 (1.0)	
Mucin summary					
Non‐mucinous	67 (44.7)	2 (22.2)	6 (21.4)	55 (55.0)	4 (30.8)
Mucinous	82 (54.7)	7 (77.8)	22 (78.6)	44 (44.0)	9 (69.2)
Not reported	1 (0.6)			1 (1.0)	

dMMR, deficient mismatch repair; hpf, high‐powered field; MHC‐I, major histocompatibility complex class‐I; pMMR, proficient mismatch repair; TILs, tumor‐infiltrating lymphocytes.

### The CRC microenvironment is composed of both conventional αβ and unconventional γδ T cells

**Figure 2:**
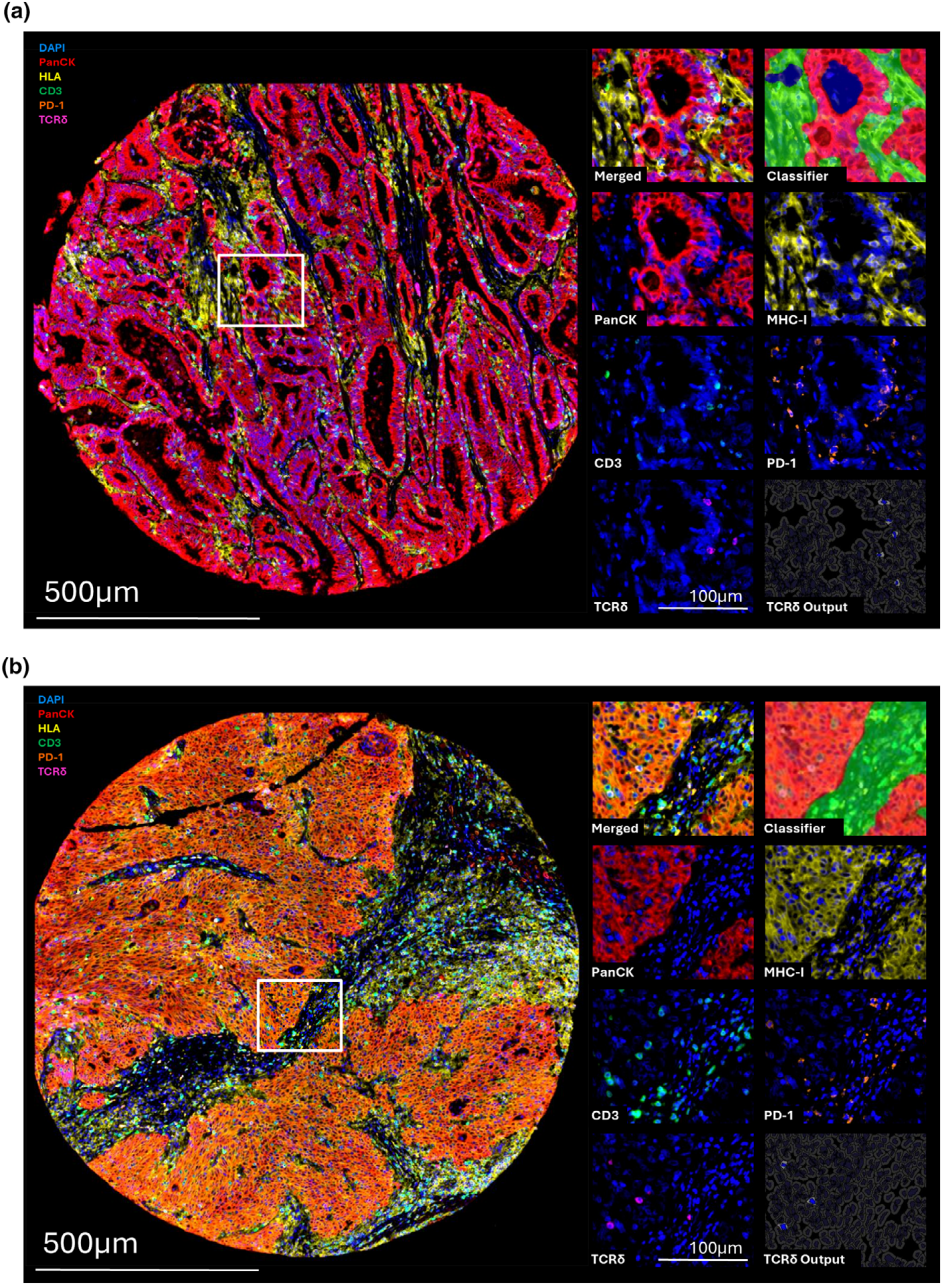
Representative examples of the tumor microenvironment of CRCs with variable MHC‐I expression. Representative CRC cores with γδ T cell infiltration and low **(a)** or high **(b)** MHC‐I expression in the tumor region. Magnified regions of interest in white boxes are represented on the right. This panel includes merged and single channel staining for PanCK, MHC‐I, CD3, PD‐1, and TCRδ with DAPI counterstains. For the same region, tumor and stromal areas recognized by the classifier are also shown (top right), alongside the analysis output of γδ T cell counts (bottom right). The scale bar indicates 500 μm on the left, and 100 μm on the right. Images were taken at 20× magnification. MHC‐I, major histocompatibility complex class I, PanCK, pan‐cytokeratin.

To investigate the impact of MHC‐I expression on immune infiltrates in the tumor microenvironment, patient cores were analysed for MHC‐I expression and immune cell presence using a custom multispectral immunohistochemistry panel. A representation of the immune cell infiltrate is illustrated using two representative cores: one with low MHC‐I expression (Figure 2a) and one with high MHC‐I expression (Figure 2b). Both cores illustrate the presence of TCRδ^+^ γδ T cells (CD3^+^ TCRδ^+^ DAPI^+^), whereas CD3^+^ T cells were decreased in the MHC‐I^−^ tumor core. To enable quantitative analysis across distinct tissue regions, classifier‐based tissue segmentation was employed to distinguish tumor (PanCK+) and stromal (PanCK‐) regions as shown in the top right of each figure. This approach enabled the precise quantification of various immune cell phenotypes across these two compartments. An example of the quantification output for TCRδ^+^ γδ T cells is included in the bottom right of each figure.

### Epithelial MHC‐I expression is consistent across stages and loss does not predict TIL scores

**Figure 3:**
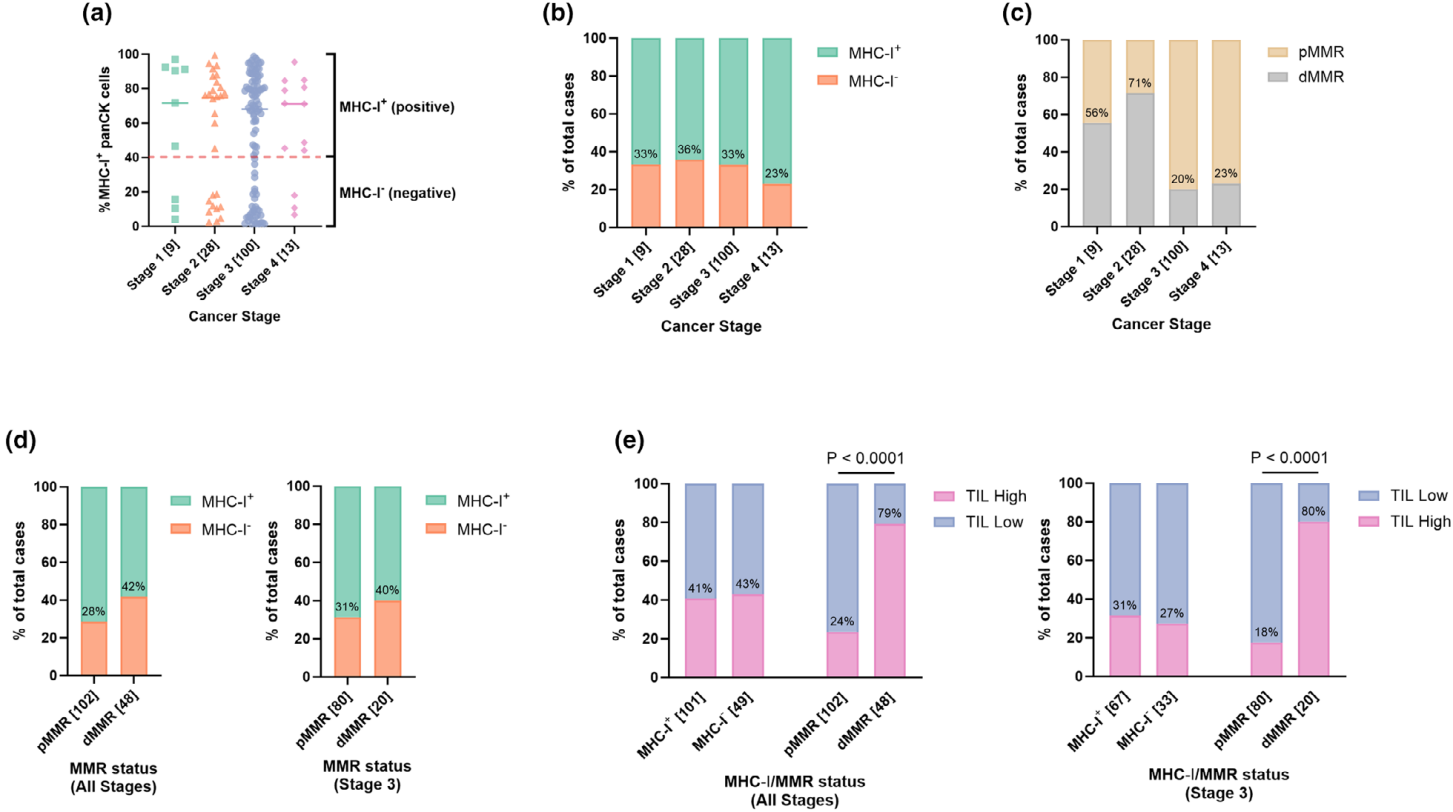
Assessment of MHC‐I expression and MMR status across CRC stages. **(a)** MHC‐I expression was quantified in individual CRC samples across stages I‐IV, where samples with less than 40% of MHC‐I^+^ PanCK^+^ cells were defined as MHC‐I negative (MHC‐I^−^). The median expression is shown. **(b)** MHC‐I and **(c)** MMR statuses are shown as a proportion of total patients in each stage. **(d)** Proportion of MHC‐I positive (MHC‐I^+^) and negative (MHC‐I^−^) tumors in pMMR or dMMR cases for all stages and stage III. **(e)** Proportion of tumors with high and low TIL scores stratified by MHC‐I and MMR status for all stages and stage III. Patient numbers for each stage and stratification are indicated in brackets. Proportions are expressed as a percentage of all patients analysed for each stage. *n* = 150 patients for all‐stage analysis, *n* = 100 patients for stage III analysis; exact proportional numbers are shown in Table 1. dMMR, deficient mismatch repair; MHC‐I, major histocompatibility complex class I; PanCK, pan‐cytokeratin, pMMR, proficient mismatch repair; TILs, tumor‐infiltrating lymphocytes. *P*‐values were calculated using two‐sided Fisher's exact tests and only *P*‐values < 0.05 were displayed. Graphs were generated using GraphPad Prism 10.

Epithelial MHC‐I expression was quantified by calculating the percentage of PanCK^+^ cells co‐expressing MHC‐I, defining tumors with MHC‐I loss as those with < 40% MHC‐I^+^ PanCK cells. This analysis demonstrated that MHC‐I expression varied across individual tumors (Figure 3a). When categorized into MHC‐I‐positive (MHC‐I^+^) or MHC‐I‐negative (MHC‐I^−^) groups, MHC‐I expression remained consistent across all CRC stages (ranging from 23 to 36% in MHC‐I^−^ tumors), with only a marginal decrease observed in stage IV samples (Figure 3b), However, not all disease stages included adequate patient numbers and thus may not be representative of the general population.

MMR status was also analysed across all CRC stages (Figure 3c). While the proportion of dMMR tumors was comparable among stage III and IV tumors (20% and 23%, respectively), there was a non‐significant trend towards a higher proportion of dMMR cases in stage I and II tumors, as expected (56% and 71%, respectively). The proportion of dMMR tumors in stage III tumors was consistent with prior reports.^10^, ^48^, ^49^

Existing literature frequently reports increased MHC‐I loss in dMMR tumors because of an increased occurrence of mutations affecting the antigen presentation pathway.^14^ The comparison of MHC‐I expression between dMMR and pMMR subgroups revealed a trend toward increased MHC‐I^−^ loss in dMMR tumors for both all‐stage (28% vs 42%, respectively) and stage III (31% vs 40%, respectively) analysis, though this did not reach statistical significance (Figure 3d).

TILs are well‐established prognostic biomarkers, particularly in neoantigen‐rich dMMR tumors, as assessed by methods including the Immunoscore.^50^ To evaluate the relationship between epithelial MHC‐I expression and overall TIL scores (as determined by a pathologist), the proportions of TIL‐high and TIL‐low tumors were analysed in both all‐stage and stage III cases based on MHC‐I status (Figure 3e). Here, no significant differences in the proportion of TIL‐high tumors were observed between MHC‐I^+^ and MHC‐I^−^ groups. In contrast, when stratifying based on MMR status, dMMR tumors were significantly enriched for TIL‐high cases compared to pMMR tumors (*P* < 0.0001 for both stage comparisons), consistent with our prior findings.^10^

### Tumor‐infiltrating γδ T cells are less abundant in advanced CRC stages

**Figure 4:**
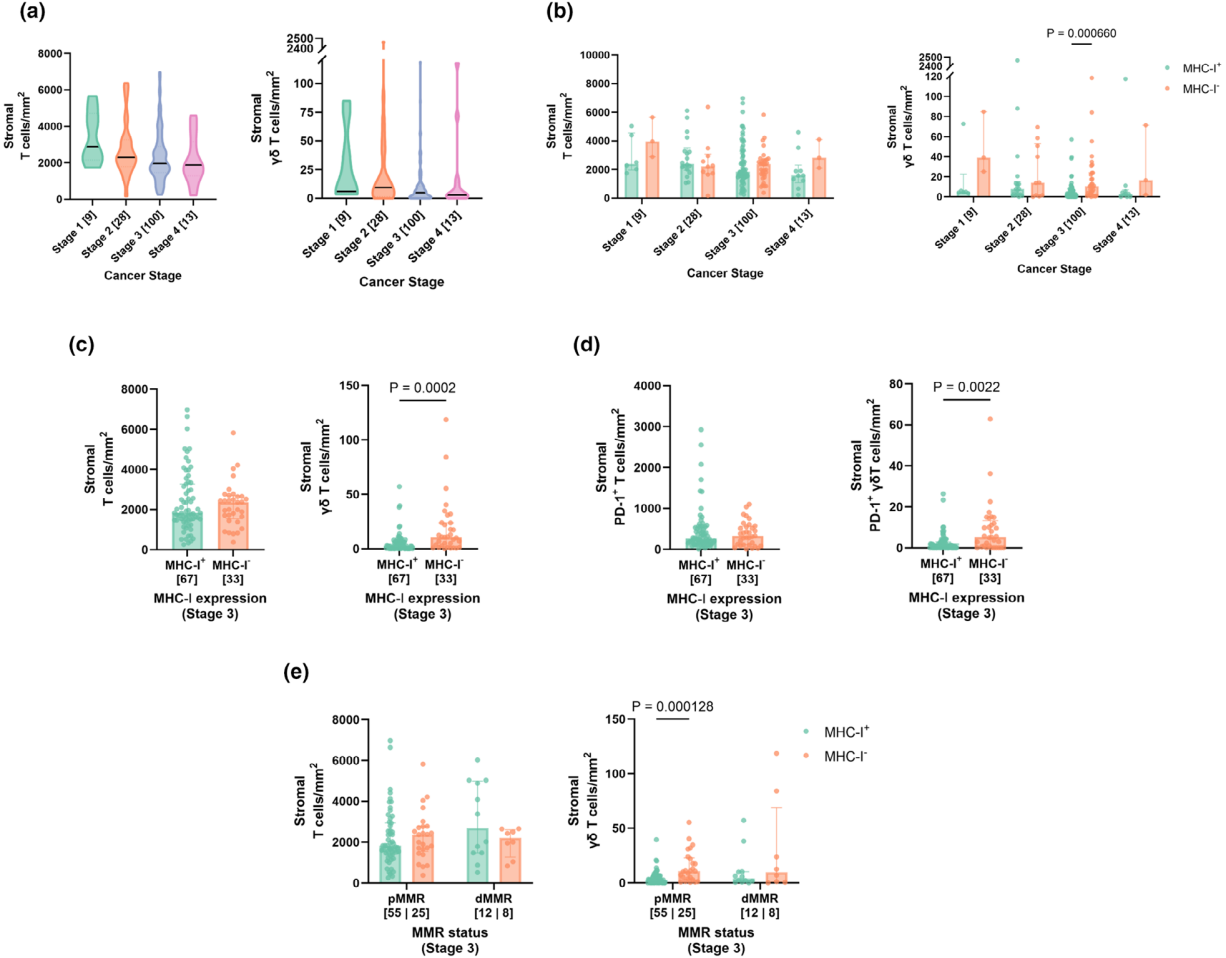
Interrogation of stromal T cells and γδ T cells in stage III CRC. **(a)** Violin plots showing the density of stromal T cells (CD3^+^ TCRδ^−^) and stromal γδ T cells (CD3^+^ TCRδ^+^) across stage I–IV CRCs. The width of the violin plot indicates the density of data points; the solid line within each violin plot represents the population median. **(b)** Density of stromal T cells (CD3^+^ TCRδ^−^) and stromal γδ T cells (CD3^+^ TCRδ^+^) in MHC‐I^+/−^ stage I‐IV CRCs. **(c)** Density of stromal T cells (CD3^+^ TCRδ^−^) and stromal γδ T cells (CD3^+^ TCRδ^+^) in MHC‐I^+/−^ stage III CRCs. **(d)** Density of PD‐1‐expressing stromal T cells (CD3^+^ TCRδ^−^ PD‐1^+^) and PD‐1‐expressing stromal γδ T cells (CD3^+^ TCRδ^+^ PD‐1^+^) in MHC‐I^+/−^ stage III CRCs. **(e)** Density of stromal T cells (CD3^+^ TCRδ^−^) and stromal γδ T cells (CD3^+^ TCRδ^+^) in MHC‐I^+/−^ pMMR or dMMR CRCs. Patient numbers for each stage and stratification are indicated in brackets. *n* = 150 patients for all‐stage analysis, *n* = 100 patients for stage III analysis. Mann Whitney U Tests were used for comparisons between two groups, with only *P*‐values < 0.05 displayed. Error bars represent the median with interquartile range of given cell densities. dMMR, deficient mismatch repair; MHC‐I, major histocompatibility complex class I, pMMR, proficient mismatch repair.

To assess the frequency and distribution of γδ T cells within tumor cores, their abundance with that of conventional T cells was compared across all stages. Three subsets were analysed for conventional T cells: tumor‐infiltrating T cells (TILs, pathologist‐scored), conventional T cells in the stroma (stromal CD3^+^ TCRδ^−^ T cells) and T cells expressing PD‐1 in the stroma (activated stromal CD3^+^ TCRδ^−^ PD‐1^+^ T cells). These populations were compared to three corresponding γδ T‐cell subsets: γδ T cells directly in the tumor region (tumor‐infiltrating CD3^+^ TCRδ^+^ γδ T cells), γδ T cells in the stroma (stromal CD3^+^ TCRδ^+^ γδ T cells) and γδ T cells expressing PD‐1 in the stroma (activated stromal CD3^+^ TCRδ^+^ PD‐1^+^ γδ T cells). A general decreasing trend in the frequency of these populations was evident across disease stages (Figure 4a, Supplementary figure 1A and B); significant reductions in TIL abundance were also observed when comparing stage II and III (*P* < 0.001) or stage II and IV tumors (*P* < 0.05) (Supplementary figure 1A).

### Stromal γδ T cells are significantly enriched in stage III pMMR CRCs with MHC‐I loss

We performed a grouped analysis across all CRC stages, comparing differences in the conventional and γδ T‐cell subsets between MHC‐I^+^ and MHC‐I^−^ CRC cores (Figure 4b, Supplementary figure 1C and D). While most T‐cell subsets showed no significant differences based on MHC‐I status, a significant increase in stromal γδ T cells was observed in stage III (*P* < 0.001) (Figure 4b) and in PD‐1‐expressing stromal γδ T cells in stages I (*P* < 0.05) and III (*P* < 0.01) (Supplementary figure 1D).

To further investigate these findings, a focussed analysis was conducted on stage III CRCs, which composed of the largest sub‐cohort with 100 cases. Here, the number of overall TILs, stromal conventional T cells and stromal PD‐1‐expressing conventional T cells did not significantly differ between MHC‐I^−^ and MHC‐I^+^ CRCs (Figure 4c and d, Supplementary figure 1E). Similarly, the number of tumor‐infiltrating γδ T cells showed no significant difference between MHC groups (Supplementary figure 1E). However, both stromal (*P* < 0.001) and PD‐1‐expressing stromal γδ T cells (*P* < 0.01) were significantly increased in CRCs exhibiting MHC‐I loss (Figure 4c and d).

Further stratification by MMR status revealed that this increase in stromal γδ T cells (*P* < 0.001) and PD‐1 expression (*P* < 0.01) was highly significant in pMMR CRCs with MHC‐I loss but not in dMMR CRCs (Figure 4e, Supplementary figure 1F). This lack of significance in dMMR was likely attributable to sample size limitations within this subgroup. Notably, the difference in total stromal conventional T cells remained insignificant for both pMMR and dMMR subsets when comparing MHC‐I^+^ and MHC‐I^−^ CRCs.

## Discussion

A quantitative immunopathology analysis employing multispectral immunohistochemistry on 150 CRC patient samples was performed to assess MHC‐I expression by calculating the proportion of PanCK+ tumor cells co‐expressing MHC‐I. Classification of tumors according to MHC‐I status requires selection of a predefined threshold despite the continuous nature of expression at a single‐cell level. The threshold used in this study was chosen to capture biologically meaningful MHC‐I loss rather than minor intratumoral heterogeneity and is consistent with prior immunohistochemical studies. This initial characterisation revealed significant heterogeneity in MHC‐I expression across various CRC stages. Nonetheless, it is important to note that alternative cut‐offs may influence classification in tumors with intermediate levels of expression. Applying a predefined threshold (defined as < 40% PanCK+ MHC‐I^+^ cells) to categorize MHC‐I‐loss (MHC‐I^−^), MHC‐I loss was detected in 23–36% of all tumors. From the available patient data within this cohort, there was a non‐significant trend towards a higher prevalence of dMMR in stage I and II CRC (56–71%) compared to stage III and IV CRC (20–23%); however, these stage‐specific dMMR proportions were intentionally enriched for dMMR cases when constructing the TMAs.

When investigating the potential correlation between dMMR status and MHC‐I loss, a non‐significant trend towards a higher proportion of MHC‐I^−^ tumors was observed within the dMMR subgroup compared to pMMR across both all‐stage and stage III comparisons. This observation is consistent with previous evidence indicating that dMMR CRC tumors frequently harbor mutations in the antigen presentation pathway affecting MHC‐I expression.^51^ Consistent with the higher mutational burden characteristic of dMMR, significantly elevated overall TIL scores were observed in dMMR tumors compared to pMMR tumors in both all‐stage and stage III analyses, with a substantially higher proportion of TIL‐high tumors in the dMMR group (*P* < 0.0001 in both comparisons). However, no significant difference was observed in the proportion of tumors with low or high TIL scores when directly comparing MHC‐I^+^ and MHC‐I^−^ populations, across either all stages or within stage III. These findings suggest that while MMR status is a robust predictor of overall immune infiltration, MHC‐I loss may influence the tumor immune microenvironment in a more nuanced manner, potentially involving differences in specific immune cell populations that are not captured by overall TIL counts.

To explore this theory, individual cell segmentation and quantification were performed for conventional T cells (CD3^+^ TCRδ^−^), γδ T cells (CD3^+^ TCRδ^+^), including PD‐1‐expressing populations of both subsets (PD‐1^+^), within both the tumor‐infiltrating and stromal compartments. Across the entire cohort, a general decreasing trend was observed for both pathologist‐defined and researcher‐scored TILs, as well as for overall stromal immune cell populations, with significant reductions in immune cell abundance noted when comparing stage II and III (*P* < 0.001) or stage II and IV (*P* < 0.05). Despite the low number of cases in stages I, II and IV, this progressive decline in overall immune infiltration at later disease stages suggests a potential compromise of immune cell effectiveness or recruitment, consistent with observations of advanced tumors excluding exhausted T cells from the tumor microenvironment.^52^

To explore whether MHC‐I^−^ tumors possess higher frequencies of other immune cell subsets, tumors were stratified by disease stage and proportions were compared across MHC‐I^+^ and MHC‐I^−^ tumors. A significant increase in stromal γδ T cells was observed in MHC‐I^−^ CRCs, a finding particularly prominent in stage III (*P* < 0.001). Furthermore, PD‐1‐expressing stromal γδ T cells were significantly increased in MHC‐I^−^ tumors in both stage I (*P* < 0.05) and stage III (*P* < 0.01). This enrichment was particularly striking in pMMR CRCs with MHC‐I loss (*P* < 0.001), suggesting that γδ T‐cell recruitment may occur independently of MMR status. While not statistically significant in dMMR CRCs because of sample size limitations, this is consistent with emerging evidence indicating that γδ T‐cell‐mediated anti‐tumor cytotoxicity can occur independently of MMR status.^53^

These results expand current understanding of γδ T‐cell involvement in CRC by demonstrating that enrichment of stromal γδ T cells in MHC‐I–deficient tumors is not restricted to dMMR CRC but is also evident in pMMR tumors, which represent the majority of CRCs.^39^ While previous studies have outlined γδ T‐cell involvement, the utilized multispectral spatial immunohistochemistry approach uniquely allowed the quantification of cellular densities within distinct tumor regions (stromal vs tumor areas). This led to the identification of significantly enriched γδ T cells specifically within the stromal compartment of MHC‐I^−^ CRCs. This spatial localisation, which is not captured by conventional bulk TIL measurements, suggests that the distribution and activation status (defined here as PD‐1 positivity) of γδ T cells is particularly crucial for their anti‐tumor function in instances of MHC‐I loss, potentially acting as a critical first line of defense or orchestrating other immune cell interactions at the tumor–stroma interface. The observed elevation of PD‐1 expression on stromal γδ T cells in MHC‐I^−^ tumors indicates active involvement in the immune response. While PD‐1 upregulation is defined here as a marker of T‐cell activation, it is also a hallmark of functional exhaustion, especially within the immunosuppressive tumor microenvironment. The dynamic activation and exhaustion status of γδ T cells remains an active area of investigation, with studies suggesting that a novel signature, including the broad expression of PD‐1 and TIGIT, is directly upregulated within the tumor microenvironment.^53^ It is thus important to note this limitation of using a mixed activation/exhaustion marker in this study.

To build upon these findings, future research should explore several avenues. A key limitation of this study is the use of a pan‐TCRδ antibody, which cannot distinguish between specific γδ T‐cell subsets (e.g. Vδ1, Vδ2, Vδ3). Despite several studies proposing the primary contribution of Vδ1 and Vδ3 subsets in CRC tissue,^39^, ^41^ their frequencies in later disease stages remains to be investigated. The cohort in this study was primarily composed of stage III tumors, a clinically and biologically relevant stage of disease in which tumor–immune interactions are particularly dynamic during the transition from localized to lymph node–positive disease. However, it is important to note that the distribution of cases across tumor stages in this cohort was uneven, with smaller numbers of stage I, II and IV tumors, which may limit statistical power for subgroup analyses in these stages. Accordingly, findings within these groups should be interpreted cautiously and considered exploratory. In contrast, the larger stage III cohort provides greater statistical robustness for the primary analyses presented here. Future studies incorporating expanded and more balanced cohorts across disease stages may further enhance statistical power for sub‐stage analyses and enable more robust correlations with disease progression. Because of the retrospective nature of this study and the inherent diversity of therapeutic interventions received by patients after surgical resections, definitive conclusions could not be drawn between immune cell infiltrate observations and survival outcomes. Assessment of a cohort of neoadjuvant ICB‐treated CRC patients may be of particular interest to assess correlations between MHC‐I loss, the γδ T cell infiltrate, and treatment responses. Finally, while this study strongly suggests a compensatory role for γδ T cells in MHC‐I^−^ tumors, direct functional validation through *in vitro* or *in vivo* studies is necessary to definitively assess the effectiveness of γδ T‐cell‐based therapies in this specific context.

## Conclusions

In conclusion, this study highlights distinctive features of tumor‐infiltrating γδ T cells in human CRC. MHC‐I loss was associated with a significant increase in the frequency and activation of stromal γδ T cells, particularly in pMMR CRCs. These findings suggest a potentially important and compensatory role for γδ T cells in tumors that have evaded conventional CD8^+^ T‐cell responses following MHC‐I loss. Collectively, these findings suggest that γδ T cells may represent an alternative immune surveillance axis in MHC‐I^−^ CRC, operating independently of MMR status. This provides a rationale to further explore γδ T‐cell‐based immunotherapeutic strategies, including adoptive cell transfer and CAR γδ T‐cell approaches, as a potential means to circumvent MHC‐I loss‐mediated immune evasion in CRC patients. Future research should focus on delineating specific γδ T‐cell subsets in CRC tumors and comprehensively interrogate their anti‐tumor capabilities, thereby contributing to current efforts investigating novel γδ T‐cell‐based strategies for the treatment of solid tumors.

## Methods

### Experimental design

A total of 192 archival primary colorectal tumor resections from patients with stage I, II, III or IV disease were retrieved for this study. These included non‐consecutive patients diagnosed at Austin Pathology between 1997 and 2013 with heterogeneous clinical follow‐up data, limiting the feasibility of a prognostic analysis. Approval for the study was granted by the Austin Health Ethics Committee (HREC/15/Austin/359) with a waiver of consent. Primary tumors from 192 CRC patients were cored and included across eight TMAs. Core number, size and tumor region specifications consisted of three 1‐mm‐diameter cores per case sampled from central tumor regions (not specifically the invasive margin). These TMAs were intentionally enriched for dMMR cases and stage III disease for the purposes of this study. Each patient tumor sample was represented by three cores, which were initially assessed for tissue integrity, adequate representation of tumor areas, staining artefacts and variability in staining. Of the 192 cases, 150 (78.1%) contained two or three intact cores suitable for downstream analysis (Figure 1a). Patients were stratified by disease stage as follows: nine in stage I, 28 in stage II, 100 in stage III and 13 in stage IV. Because of uneven case numbers across tumor stages, stage‐stratified analyses for stage I, II, and IV tumors were considered exploratory. The larger stage III subgroup enabled more robust statistical comparisons and therefore formed the primary focus of the stage‐specific analyses. Histopathological and clinical data were obtained and integrated with the staining data generated in this study (Figure 1b). Detailed cohort characteristics, including demographics and clinical features, are outlined in Table 1. Tumor‐infiltrating lymphocyte (TIL) counts were initially derived from haematoxylin and eosin (H&E)‐stained whole tumor sections, as previously described.^48^ TILs were scored based on infiltration into the tumor epithelium, with a stringent exclusion of lymphocytes found in the stroma or within lymphoid aggregates. Tumors were then classified as having a high TIL count (> 10 lymphocytes per 5 high‐powered fields, hpfs) or a low TIL count (< 10 lymphocytes per 5 hpfs). The five representative hpfs for each sample were randomly selected and scored by an independent pathologist, blinded to clinicopathological data. TIL cell counts were normalized to cells/mm^2^ for cell density analysis in this study. Analysis of DNA mismatch repair status was conducted by immunohistochemistry for nuclear expression of MLH1, PMS2, MSH2 and MSH6, as previously described.^48^

### Multispectral immunohistochemistry

Sections from eight TMAs were stained for the expression of CD3 (Sigma‐Aldrich, HPA043955, polyclonal, 1:250 dilution), TCRδ (Santa Cruz, Sc‐100 289, H‐41 clone, 1:100 dilution), PD‐1 (Abcam, Ab52587, NAT105 clone, 1:100 dilution), MHC‐I (kindly provided by Brian Tait, Victorian Transplantation and Immunogenetics Service, Melbourne, Australia, preferentially recognizes beta‐2‐microglobulin‐free HLA‐A, HLA‐B, HLA‐C heavy chain, HC10 clone, 1:3000 dilution)^54^, ^55^ and pan‐cytokeratin (PanCK, Sigma‐Aldrich, C2562, C‐11 + PCK‐26 + CY‐90 + KS‐1A3 + M20 + A53‐B/A2 clones, 1:250 dilution) (Supplementary table 1), using a modified previously described Opal^TM^‐TSA multispectral immunohistochemisty panel (modifications included removing granzyme B, BTN2A1 and BTN3A1, while adding PD‐1 and MHC‐1 to address a different research question).^56^ This modified panel was initially optimized using the Opal™ Multiplex IHC Assay Development Guide and Image Acquisition Information (Akoya Biosciences). This optimisation standard procedure, which included adequate tissue and staining controls (positive, negative and autofluorescence), single colour controls, number of heat‐induced epitope retrievals, antigen retrieval pH selection, primary antibody‐Opal™ pairing and order, staining location and intensity, has been previously described in detail.^56^ When staining the eight TMAs, slides were baked at 65°C for 1 h, dewaxed in xylene thrice for 10 min, rehydrated in ethanol twice for 10 min and stained manually. Following rehydration, endogenous peroxidases were blocked with BLOXALL® (Vector Labs, SP‐6000) for 10 min. Multiple rounds of heat‐induced epitope retrieval were performed for 15 min using AR9 buffer (Akoya Biosciences®, AR9001KT, 1X), non‐specific binding site blocking for 15 min (Akoya Blocking/Antibody Diluent [Akoya Biosciences®, ARD100]), a 30‐min incubation with primary antibody (MHC‐I, PD‐1, TCRδ, CD3 or PanCK in this particular order), a 30‐min secondary antibody (ImmPRESS® HRP Universal [Horse Anti‐Mouse/Rabbit IgG] PLUS Polymer [Vector Labs, MP‐7800‐15]) incubation and a 10‐min TSA incubation using Opal™ fluorophores (Opal™ 520 [1:100 dilution], 570 [1:100 dilution], 620 [1:100 dilution], 650 [1:100 dilution] or 690 [1:50 dilution] [Akoya Biosciences®, FP1487001KT, FP1488001KT, FP1495001KT, FP1496001KT, FP1497001KT, respectively], in this particular order) diluted in Akoya Plus Amplification Diluent (Akoya Biosciences®, FP1498) to detect all target proteins. Slides were counterstained for 5 min with spectral DAPI (Akoya Biosciences®, FP1490, 1X), mounted using VECTASHIELD Vibrance® Antifade Mounting Medium (Vector Labs, H‐1700‐2) and cover‐slipped. Whole slide scan images were captured at 10× magnification using the Vectra 3 Automated Quantitative Pathology Imaging System. Individual cores for each TMA were selected using the TMA function in Phenochart Whole Slide Viewer (Akoya Biosciences®). Regions of interest spanning the entirety of each tissue specimen were captured at 20× magnification, spectrally unmixed using an appropriate spectral library on the inForm Tissue Analysis software (both Akoya Biosciences) and stitched into single QPTIFF files using the HALO Image Analysis software (Indica Labs).

### Tissue segmentation, cell phenotyping and quantification

Frequency of immune cell subsets and MHC‐I expression were quantified using the HALO Image Analysis software (Indica Labs). Tissue classifier masks for stromal, tumor or empty regions were created using Random Forest machine‐learning algorithms. Each core had an individual tissue classifier mask created using annotated regions from the same tissue. Tissue classifiers were trained using approximately 100 annotated regions per TMA and applied on a patient‐by‐patient basis (2 or 3 cores at a time), after which a visual quality assessment was performed. Tissue classifier masks were then analysed using the Indica Labs HighPlex FL v4.2.14 module based on nuclear detection using DAPI (AI Nuclei Seg—FL v1), followed by membrane and cytoplasm detection to approximate whole‐cell boundaries. Marker expression was quantified using single‐cell segmentation and intensity measurement within the image analysis platform (single‐cell classification). A positivity threshold for each marker was defined based on signal intensity to distinguish true cytoplasmic and/or membranous staining from autofluorescence or low‐level background signal. Marker signal intensities above this threshold were classified as positive, while those below the threshold were classified as negative. Cell phenotyping was performed to individually quantify CD3^+^ TCRδ^−^ (T cells), CD3^+^TCRδ^+^ (γδ T cells), CD3^+^ TCRδ^−^ PD‐1^+^ (activated T cells), CD3^+^ TCRδ^+^ PD‐1^+^ (activated γδ T cells) and PanCK^+^ (tumor cells) populations relative to classifier regions. If any mis‐segmented regions or misclassified cells were identified upon manual curation, these were corrected by either refining the classifier with additional regions or adjusting the positive signal thresholds using real‐time tuning. To accurately quantify the number of CD3^+^TCRδ^−^ cells (non‐γδ T cells), the number of CD3^+^TCRδ^+^ cells was subtracted from the total CD3^+^ count, and the number of CD3^+^TCRδ^+^PD‐1^+^ cells was subtracted from the total CD3^+^PD‐1^+^ count. CD3^+^ TCRδ^+^ TILs were quantified as CD3^+^ TCRδ^+^ cells located within PanCK^+^ tumor regions. Each cell phenotype was quantified across the classified stromal and tumor regions.

The quantification of MHC class I (MHC‐I) expression in tumor tissue remains an area of active discussion with different methodologies proposed by several groups,^16^, ^17^, ^18^, ^19^, ^20^, ^21^, ^22^, ^23^, ^24^ including multispectral immunohistochemistry.^25^ Tumor cells were classified as MHC‐I positive or negative based on the positivity threshold, acknowledging that MHC‐I expression exists along a continuum. For each tumor, the proportion of tumor cells classified as MHC‐I positive was calculated (tumor‐level classification). Here, a binary classification of MHC‐I expression was used, similar to the method utilized by Kaneko *et al*.^22^ where tumors were classified as exhibiting MHC‐I loss when the proportion of MHC‐I–positive tumor cells fell below a predefined 40% threshold (< 40% PanCK+ MHC‐ cells, MHC‐I negative [MHC‐I^−^]), as illustrated in Figure 1c. This threshold was selected to identify tumors with widespread MHC‐I loss rather than focal spatial heterogeneity in MHC‐I expression that may occur within otherwise positive tumors (> 40% PanCK+ MHC‐ cells, MHC‐I positive [MHC‐I^+^]).

### Statistical analysis

All statistical analyses were conducted using the GraphPad Prism 10 software (Dotmatics). Data distributions were assessed for normality and found to be nonparametric. Therefore, nonparametric statistical tests were used for all comparisons. The Mann–Whitney U test was used to compare differences between two independent groups. For comparisons involving three or more independent groups, the Kruskal–Wallis test (nonparametric one‐way ANOVA) was performed. A *P*‐value of < 0.05 was considered statistically significant.

## Author contributions

**Luke T Quigley:** Investigation; methodology; writing – review and editing. **Jessica Da Gama Duarte:** Conceptualization; resources; supervision; methodology; funding acquisition; writing – original draft; writing – review and editing. **Andreas Behren:** Conceptualization; resources; supervision; funding acquisition; writing – review and editing. **Kok Fei Chan:** Writing – original draft; writing – review and editing. **Tianming A Li:** Investigation; writing – original draft; writing – review and editing. **David S Williams:** Resources; writing – review and editing. **Lisa A Mielke:** Resources; supervision; writing – original draft; writing – review and editing.

## Conflict of interest

The authors declare no conflict of interest.

## Ethics approval and consent to participate

Ethics approval for the study was granted by the Austin Health ethics committee (HREC/15/Austin/359) with a waiver of consent.

## Supporting information


Supplementary data 1

## Data Availability

The data that support the findings of this study are available from the corresponding author, JDGD, upon reasonable request.
