# Supplementary material for: Characterisation of tumor‐infiltrating gamma‐delta T cells in human colorectal cancer with MHC‐I loss
Source: Clin Transl Immunology. 2026 May 12;15(5):e70097. doi: 10.1002/cti2.70097 (PMC13161889; doi:10.1002/cti2.70097)
Supplement: Supplementary file 1 — Supplementary data 1 [file CTI2-15-e70097-s001.docx]

Supplementary Materials for

**Characterization of tumor-infiltrating γδ T cells in human colorectal cancer with MHC class-I loss**

Tianming Li et al.

*Corresponding author. Email: jessica.dagamaduarte@monash.edu

**This file includes:**

Fig. S1

Table S1


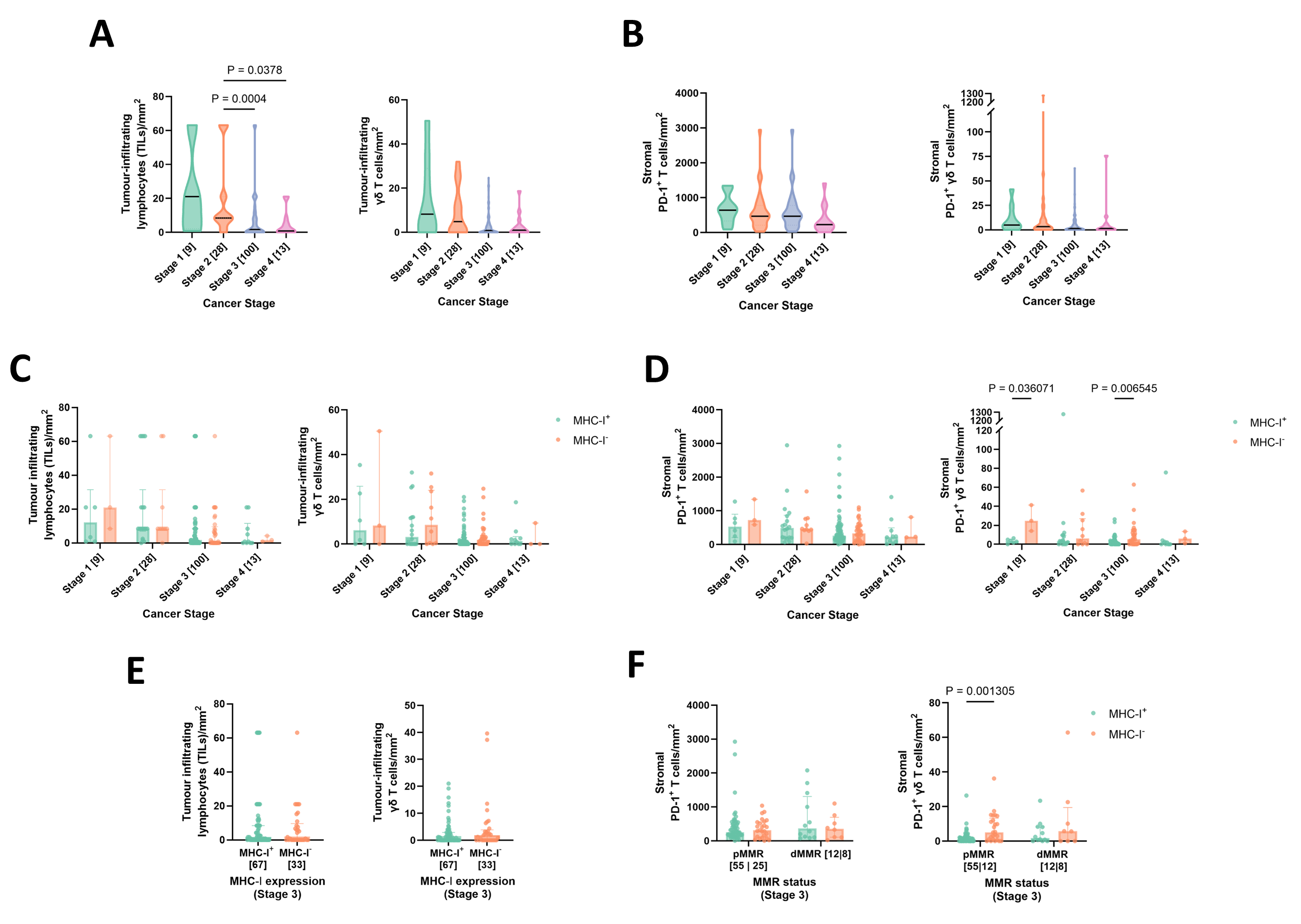


Fig. S1. Interrogation of stromal and tumor-infiltrating T cells and γδ T cells in CRC

(**A**) Violin plots showing the density of TILs (pathologist scored) and tumor-infiltrating γδ T cells (CD3^+^ TCRδ+, researcher scored) across stage I-IV CRCs. (**B**) Violin plots showing the density of PD-1 expressing stromal T cells (CD3^+^ TCRδ^-^ PD-1^+^), and PD-1-expressing stromal γδ T cells (CD3^+^ TCRδ^+^ PD-1^+^) across stage I-IV CRCs. The width of the violin plot indicates the density of data points, the solid line within each violin plot represents the population median. (**C**) Density of TILs (pathologist scored) and tumor-infiltrating γδ T cells (CD3^+^ TCRδ^+^, researcher scored) in MHC-I^+/-^ stage I-IV CRCs. (**D**) Density of PD-1-expressing stromal T cells (CD3^+^ TCRδ^-^ PD-1^+^) and PD-1-expressing stromal γδ T cells (CD3^+^ TCRδ^+^ PD-1^+^) in MHC-I^+/-^ stage I-IV CRCs. (**E**) Density of TILs (pathologist scored) and tumor-infiltrating γδ T cells (CD3+ TCRδ+, researcher scored) in MHC-I+/- stage III CRCs. (**F**) Density of PD-1-expressing stromal T cells (CD3+ TCRδ-) and PD-1-expressing stromal γδ T cells in (CD3+ TCRδ+) in MHC-I+/- pMMR or dMMR CRCs. Patient numbers for each stage and stratification are indicated in brackets. n = 150 patients for all-stage analysis, n = 100 patients for stage III analysis. Mann Whitney U Tests were used for comparisons between two groups, with only p-values < 0.05 displayed. Error bars represent the median with interquartile range of given cell densities. MHC-I: major histocompatibility complex class I, pMMR: proficient mismatch repair, dMMR: deficient mismatch repair.

| **REAGENT or RESOURCE** | **SOURCE** | **IDENTIFIER** |
| --- | --- | --- |
| **Antibodies** |  |  |
| Mouse monoclonal anti-human MHC Class I antibody | ONJCRI, in house | N/A (1:3000 dilution) |
| Mouse monoclonal anti-human PD-1 [NAT105] | Abcam | Ab52587(1:100 dilution) |
| Mouse monoclonal anti-human TCRδ [H-41] | Santa Cruz Biotechnology | Sc-100289 (1:100 dilution) |
| Rabbit polyclonal anti-human CD3e [00003625] antibody | Merck | HPA043955 (1:250 dilution) |
| Mouse monoclonal anti-human Anti-Cytokeratin [C-11+PCK-26+CY-90+KS-1A3+M20+A53-B/A2] | Merck (Sigma-Aldrich) | C2562 (1:100 dilution) |
| ImmPRESS® HRP goat anti-rat IgG mouse adsorbed Polymer Detection Kit, Peroxidase | Vector Laboratories | Cat#VEMP744415 |
| ImmPRESS® HRP horse anti-rabbit IgG Polymer Detection Kit, Peroxidase | Vector Laboratories | Cat#VEMP740150 |
| **Biological samples** |  |  |
| Human colorectal cancer FFPE tumour microarrays, male and female (22-94 years old) | Associate Professor David Williams, Austin Health | N/A |
| **Critical commercial assays** |  |  |
| Opal 6-plex Manual Detection Kit* | Akoya Biosciences | Cat#NEL811001KT |
| **Software and algorithms** |  |  |
| Phenochart Whole Slide Viewer | Akoya Biosciences | <https://www.akoyabio.com/support/software/> |
| inForm® Tissue Analysis Software | Akoya Biosciences | <https://www.akoyabio.com/support/software/> |
| HALO® Image Analysis Platform | Indica Labs | <https://indicalab.com/halo/> |
| **Other** |  |  |
| Vectra® 3 Automated Quantitative Pathology Imaging System | Akoya Biosciences | <https://www.akoyabio.com/phenoimager/instruments/vectra-3-0/> |

Table S1. Materials table

*Includes antigen retrieval (AR) buffer at pH6 (AR6 buffer), AR9 buffer, antibody diluent and blocking buffer formulation (Blocking/Antibody Diluent), Opal™ Polymer horseradish peroxidase (HRP) mouse and rabbit (reacts specifically with primary antibodies raised in mouse and rabbit), Opal™ signal amplification diluent (1× Plus Amplification Diluent), 6 reactive Opal™ fluorophores (Opal™ 520, 540, 570, 620, 650 and 690), and spectral DAPI
